# Inflammation-associated gene expression is altered between normal human ovarian surface epithelial cells and cell lines derived from ovarian adenocarcinomas

**DOI:** 10.1038/sj.bjc.6602568

**Published:** 2005-05-03

**Authors:** O Gubbay, W Guo, M T Rae, D Niven, S P Langdon, S G Hillier

**Affiliations:** 1 Centre for Reproductive Biology, The Chancellor's Building, University of Edinburgh, 49 Little France Crescent, Old Dalkeith Road, Edinburgh EH16 4SB, UK; 2 Cancer Research UK, Edinburgh Oncology Unit, Western General Hospital, Edinburgh EH4 2XU, UK

**Keywords:** ovarian cancer, ovarian surface epithelium, 11*β*HSD-1/2, inflammation

## Abstract

Ovulation is believed to contribute to the development of ovarian cancers that derive from the ovarian surface epithelium (OSE). The process of ovulation is synonymous with inflammation and inflammatory cytokines such as interleukin-1*α* (IL-1*α*) have recently been shown to induce both inflammatory and anti-inflammatory responses in human OSE (HOSE) cells. In this study we directly compared levels of IL-1*α*-induced gene expression by analysing the levels of 11*β*-hydroxysteroid dehydrogenase (11*β*HSD) types 1 (11*β*HSD-1) and 2 (11*β*HSD-2), cyclooxygenase-2 (COX-2), IL-1 receptor (IL-1R) and glucocorticoid receptor *α* (GR*α*) mRNA between normal HOSE cells and cell lines derived from poorly differentiated (SKOV-3, BG-1, PEO-4) and well-differentiated (PEO-14) ovarian adenocarcinoma. In HOSE cell cultures, and to a lesser extent PEO-14 cells, the basal mRNA levels of COX-2 and 11*β*HSD-1 were relatively high and further shown to be induced in response to IL-1*α* (for HOSE cells; >20-fold, *P*<0.05 and PEO-14 cells; >3fold, *P*<0.05). However, whereas HOSE cells expressed a low level of 11*β*HSD-2 mRNA that was only mildly responsive to IL-1*α* (1.3-fold, *P*<0.001), all cell lines exhibited a higher basal level of 11*β*HSD-2 mRNA that was in some cases further stimulated in PEO-4 cells (five-fold; *P*<0.05) or suppressed in SKOV-3 cells (two-fold; *P*<0.01) in response to IL-1*α*. All cells tested expressed IL-1R and, with the exception of BG-1, GR*α*. These results indicate that cell lines derived from ovarian cancers have lost the ability to respond normally to inflammatory cytokines such as IL-1*α*. The finding that normal OSE cells, in contrast to cell lines derived from patients with ovarian adenocarcinoma, abundantly express 11*β*HSD-1 mRNA but are essentially devoid of 11*β*HSD-2 mRNA supports the concept that the pattern of 11*β*HSD isoform gene expression is a defining feature of neoplastic cellular transformation, which might have particular relevance to the ovary.

The surface of the ovary is covered by a continuous layer of cuboidal epithelial cells known as the ovarian surface epithelium (OSE). Although human OSE (HOSE) cells have been collected, cultured and studied *in vitro*, their natural function beyond forming a physical barrier between ovary and other peritoneal surfaces is unclear (Auersperg *et al*, 2001). The OSE cell layer, however, would appear to be important for ovulation since ovulation does not proceed in its absence (Colgin and Murdoch, 1997). Repeated episodes of ovulation-associated injury and repair are presumed to underlie the high frequency of ovarian carcinoma arising from the OSE (Fathalla, 1971; Salazar *et al*, 1996), which account for 90% of all ovarian cancers (Ozols, 1991). Since ovulation is a natural inflammatory process (Espey, 1980a, 1980b), factors related to inflammation of the OSE have been associated with increased risk of ovarian cancer (Ness and Cottreau, 1999; Ness *et al*, 2000). It is therefore critically important to understand how inflammatory cell damage is normally resolved in the OSE.

The inflammatory response at ovulation induces extensive tissue remodelling (Murdoch *et al*, 1999; Bukulmez and Arici, 2000) and accompanying cell death that is observed within OSE cells (Murdoch *et al*, 1999). Mutations responsible for generating cancerous cells are believed to arise from DNA replication/repair errors during subsequent rounds of OSE cell proliferation. The rate of mutation is further believed to increase in the presence of toxic oxidants that are released during such an inflammatory response (Ness and Cottreau, 1999).

In patients with ovarian cancer, increased levels of IL-1 and IL-1R are present in serum (Zeisler *et al*, 1998; Kondera-Anasz *et al*, 2003). These levels, however, are suggested to arise as a consequence of ovarian cancer, rather than playing any causal role (Punnonen*et al*, 1991; Kutteh and Kutteh, 1992). This is further exemplified by the positive effect of IL-1*α* treatment in patients with ovarian cancer (Buescher *et al*, 1993; Lee *et al*, 1993; Vadhan-Raj *et al*, 1994; Verschraegen *et al*, 1996). A closer look at the IL-1*α* gene in cancer patients showed no association with polymorphisms (Hefler *et al*, 2002). However, polymorphism of the interleukin receptor antagonist (IL-1RA) gene was shown to be associated with ovarian cancer (Sehouli *et al*, 2003a, 2003b, 2002). In patients with ovarian cancer, IL-1RA is present at increased levels (Burger *et al*, 1994; Fujiwaki *et al*, 1997; Kondera-Anasz *et al*, 2003). More detailed studies on the role of IL-1*α* in ovarian cancer have produced conflicting results. Thus, while IL-1 is reported to increase ovarian cancer cell growth in an autocrine fashion (Marth *et al*, 1996; Huleihel *et al*, 1997; Kawakami *et al*, 1997), IL-1 is documented to enhance ovarian cancer cell death and inhibit cellular repair mechanisms (Benchekroun *et al*, 1995; Wang *et al*, 1996).

Interleukin-1, the prototypical inflammatory cytokine known to be produced in association with ovulation is documented to stimulate inflammatory changes in primary HOSE cell cultures, measured as increased gene expression of cyclooxygenase-2 (COX-2) (Rae *et al*, 2004) and matrix metalloproteinases (Niven *et al*, 2004). IL-1*α* also simultaneously induces increased expression and activity of 11*β*-hydroxysteroid dehydrogenase type 1 (11*β*HSD-1) in HOSE cells (Yong *et al*, 2002). 11*β*HSD-1 is a steroid dehydrogenase/reductase enzyme that reversibly promotes increased formation of cortisol from substrate cortisone (Tannin *et al*, 1991; Stewart and Mason, 1995). The local increase of cortisol is believed to play a role in providing an anti-inflammatory environment to counteract inflammation caused at ovulation (Andersen and Hornnes, 1994; Escher *et al*, 1997; Hillier and Tetsuka, 1998; Yong *et al*, 2002; Rae *et al*, 2004). In addition to 11*β*HSD-1, an additional 11*β*HSD isoform, 11*β*HSD-2, converts cortisol to cortisone; reviewed in Michael *et al* (2003). Although inflammatory stimulation appears not to alter expression of 11*β*HSD-2 in HOSE cells (Rae *et al*, 2004), an increased level of 11*β*HSD-2 is present within tumors that originate from breast, colon, adipose, adrenal and pituatory tissue (Rabbitt *et al*, 2003).

Here we directly compared the levels of gene expression of COX-2, 11*β*HSD-1 and -2 in response to treatment with IL-1*α* in normal HOSE cells and a series of cell lines derived from the ovaries of patients with ovarian adenocarcinoma (Langdon *et al*, 1988).

## MATERIALS AND METHODS

### Isolation of HOSE cells

HOSE cells were obtained with informed consent after local ethical committee approval from the ovaries of premenopausal women undergoing elective surgery for nonmalignant gynaecological conditions. Cells were collected at laparotomy by gentle scraping of the ovarian surface with a sterile wooden spatula, which was then rinsed into sterile, warmed HOSE1 culture media (see below). Cells were collected as near to the beginning of surgical procedure as practicable to avoid any contamination with blood cells. Collections were then examined by phase-contrast microscopy to ensure that sufficient flakes of OSE had been obtained and cultured in donor calf serum-precoated flasks (75 cm^2^, Corning Inc., Glass Works, Corning NY, USA).

### Culture of HOSE cells

Culture media (HOSE 1) consisted of Medium199:MCDB105 (1 : 1 v v^−1^) supplemented with foetal calf serum (15%v v^−1^), streptomycin (50 *μ*g ml^−1^), penicillin (50 IU ml^−1^) and L-glutamine (1 mmol l^−1^) (8). Cells were incubated at 37°C in a humidified incubator under an atmosphere of 95% air, 5% CO_2_ for up to 28 days, with media renewed every 7 days. Confluent cell monolayers were routinely obtained in 21 days using this system. Monolayers were routinely examined by phase-contrast microscopy for contaminating cells such as fibroblasts, and confirmation of cell purity was confirmed in selected cases with immunocytochemical staining for cytokeratin 7, 8, 18 and 19 (Czernobilsky, 1985; van Niekerk *et al*, 1991) using a commercially available monoclonal anti-human cytokeratin antibody (Dako), which revealed that monolayers were pure epithelial cells using this culture system (data not shown). Confluent HOSE monolayers were treated with trypsin-EDTA in Hanks balanced salt solution (0.05% w v^−1^ trypsin, 0.5 mM EDTA, Invitrogen) at 37°C for 5 min. Cells were then collected by centrifugation at 800 **g** for 5 min. This pellet was washed in fresh HOSE 1 media, and then resuspended in fresh media. Cell number and viability were determined by trypan blue (Sigma) exclusion counting in a haemocytometer; viability ranged from 75 to 95%. HOSE cells were seeded in six-well plates (50 000 well^−1^) and incubated with HOSE 1 medium for 48 h. For treatments, HOSE cells were incubated for a further 24 h in Dulbecco's modified Eagle's medium nutrient F-12, containing 100 U ml^−1^ penicillin, 100 *μ*g ml^−1^ streptomycin (as used for cell lines) and subsequently treated with 0.25, 0.5 or 1 ng ml^−1^ IL-1*α* (R&D Systems Europe Ltd, Abingdon, Oxon, UK) for a further 48 h. All tissue culture reagents were obtained from Gibco BRL (Life technologies Ltd, Renfrewshire, UK) and Sigma Chemical Co. (Poole, Dorset, UK).

### Cell lines

The following cell lines SKOV-3, BG-1, PEO-4 and PEO-14 were kindly provided by P Pujol, INSERM, Montpellier, France. Cells were routinely grown in complete medium (Dulbecco's modified Eagle's medium nutrient F-12, containing 100 U ml^−1^ penicillin, 100 *μ*g ml^−1^ streptomycin and 10% foetal calf serum). The cell lines were seeded in six-well plates (50 000 cells well^−1^) and incubated with serum-free medium for 24 h. The cells were subsequently treated with 0.25, 0.5 or 1 ng ml^−1^ IL-1*α* (R&D Systems Europe Ltd, Abingdon, Oxon, UK) for a further 48 h.

### RNA extraction and quality analysis

RNA was extracted from HOSE cells using RNeasy minispin columns (Qiagen) as per manufacturer's protocol. In total, 1 *μ*l aliquots of purified RNA were removed for quantification and quality assessment. RNA was quantified and quality assessed using the Agilent 2100 Bioanalyser system for total RNA in combination with RNA6000nano chips (Agilent Technologies, Cheshire, UK). Only RNA that displayed intact 18S and 28S peaks was reverse transcribed to cDNA for PCR analysis. This quality control step was included for each experimental run to avoid generation of false negative results due to RNA degradation prior to and during extraction steps, and also as a quantification method to ensure equal amounts of RNA were transcribed in each RT- reaction.

### Real-time PCR analysis

Total RNA (1 *μ*g) was treated with DNAase (Invitrogen) as per the manufacturer's protocol. In total, 200 ng treated RNA was reverse transcribed (random hexamer kit; Applied Biosystems), and 2 *μ*l of the RT-mix was analysed. cDNA was analysed in a 25 *μ*l final volume assay system containing 300 nmol l^−1^ primers and 200 nmol l^−1^ TaqMan hybridisation probe (Biosource UK Ltd). Primers and probes were designed using Primer-Express software (Perkin-Elmer), where possible spanning intron regions to avoid any poterntial of genomic DNA amplification. For human 11*β*HSD-1, the following was used: forward primer (AGG ATC TTC CTG CAT GGA TTT C), reverse primer (AGC TCT GCG CCA AGA AGA AGT) and probe (TGA CAG CTC ACT CTG GAC CAC TCT TCT GA). For human 11*β*HSD-2, the following was used: forward primer (GGC CAA GGT TTC CCA GTG A), reverse primer (GTT GTG CCA GGA GGG TGT TT) and probe (CTC TGC GCC TCT CCA CTG TTT CAT GA). For human COX-2, the following was used: forward primer (CCT TCC TCC TGT GCC TGA TG), reverse primer (ACA ATC TCA TTT GAA TCA GGA AGC T) and probe (TGC CCG ACT CCC TTG GGT GTC A). For human IL-1R (type 1), the following was used: forward primer (TGT CAC CGG CCA GTT GAG T), reverse primer (GCA CTG GGT CAT CTT CAT CAATT) and probe (ACA TTG CTT ACT GGA AGT GGA ATG GGT CAG). Target mRNA was quantified in relation to 18S ribosomal RNA abundance in each sample, with suitable positive control RNA (human liver total RNA from Ambion and in house prepared human placental mRNA). Negative controls included RT-negative samples (RNA template with no reverse transcriptase enzyme), RT-H_2_O (water in place of RNA template) samples generated at the time of reverse transcription of samples and a Taqman reaction negative control where cDNA was replaced with water. Primer and probe sets for COX-2 were the kind gifts of Dr H Jabbour (HRSU, Medical Research Council, Edinburgh, UK). Data are presented relative to 18S as mean±s.e.m. and statistics performed using the superANNOVA package from Abacus Concepts, Inc.

### Analysis of RT–PCR by agarose gel electrophoresis

Reverse transcription reactions were conducted in a volume of 50 *μ*l consisting of 2 *μ*g of total RNA, 10 ng oligo-dT (Gibco Invitrogen Corporation), 0.01 M DTT, 1 mM dNTPs and Superscript reverse transcriptase (Gibco Invitrogen Corporation). PCR was performed in 25 *μ*l reactions, containing 2 *μ*l of the reverse transcription reaction with reagents provided by Hybaid UK including 10% dimethyl sulphoxide. PCR samples were heated at 94°C for 2 min, followed by 30 cycles of 94°C for 30 s, 55°C for 30 s and 72°C for 30 s, and an extension time of 5 min at 72°C. The following primers were used in PCR reactions (20 pmol per reaction): human IL-1 (type 1) receptor (forward: ATC TAC AGA ACA AGC CTC CAG G and reverse: CCA CAC TGT AAT AGT CTT CC), human 11*β*HSD-1 (forward: TGT AGG TTC TCT CTG TGT GTC C and reverse: GCA AAT GTT AGA GGA ACT CC), human 11*β*HSD-2 (forward: GTA TTG GAG TTG AAC AGC CCC G and reverse: AGA GAC ACT TGG GAT TTA GCC C) and human COX-2 (forward: CGA GGT GTA TGT ATG AGT GTG G and reverse: GCA ATC ATC AGG CAC AGG), human GR*α* sense (forward: ACA CAG GCT TCA GGT ATC TT and reverse: ACT GCT TCT GTT GCC AAG). PCR fragments were visualised by ethidium bromide staining on 1% agarose gels.

## RESULTS

### mRNA analysis of cancer cell lines and HOSE cells

**Figure 1:**
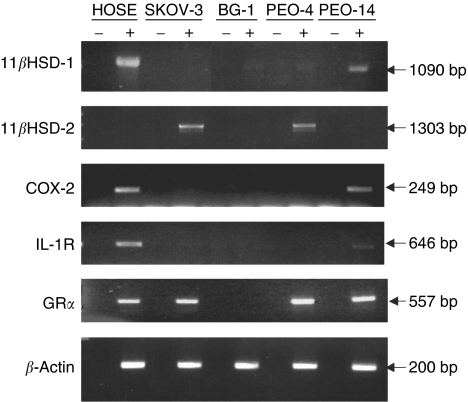
RT–PCR of 11*β*HSD-1 and -2, COX-2, IL-1 receptor, GR*α* and *β*-actin using RNA isolated from HOSE, SKOV-3, BG-1, PEO-4 and PEO-14 cells. Reactions were performed in the absence (−) and presence (+) of reverse transcriptase. One example of three separate experiments is presented.

**Figure 2:**
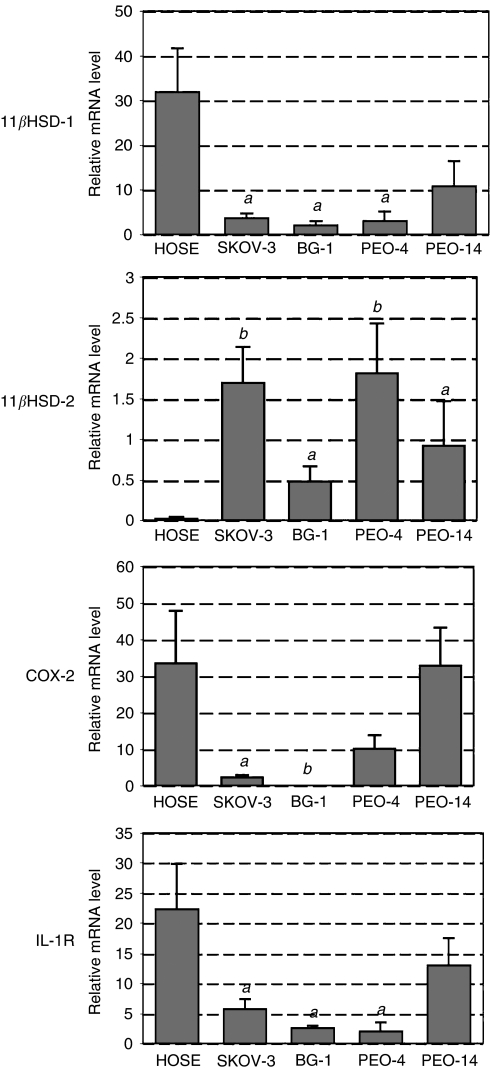
Real-time PCR using RNA isolated from HOSE, SKOV-3, BG-1, PEO-4 and PEO-14 cells. Level of mRNA was standardised to 18S using an internal control and each sample normalised to a reference sample. Data (*n*=4) are presented as fold increase and mean±s.e.m. Letters denote significance relative to HOSE cells: *a*: *P*<0.01 and *b*: *P*<0.001.

In order to compare levels of gene expression between HOSE cells and cancer cell lines, RNA was isolated and analysed by RT–PCR either by agarose gel electrophoresis (Figure 1) or real-time PCR (Figure 2). For HOSE cells, levels of 11*β*HSD-1, COX-2, IL-1 receptor (IL-1R) and glucocorticod receptor *α* (GR*α*) mRNA were easily detected; however, 11*β*HSD-2 mRNA was barely detected. In contrast, all four cancer cell lines showed significantly lower levels of 11*β*HSD-1 mRNA but higher levels of 11*β*HSD-2, relative to HOSE cells. Whereas PEO-14 cells exhibited patterns of COX-2 and IL-1R mRNA expression similar to that of HOSE cells, these two genes were expressed at much lower levels in SKOV-3, BG-1 and PEO-4 cells. With the exception of BG-1 cells, GR*α* mRNA was readily detected in all cells.

### Effect of IL-1*α* on 11*β*HSD-1 mRNA in cancer cell lines and HOSE cells

**Figure 3:**
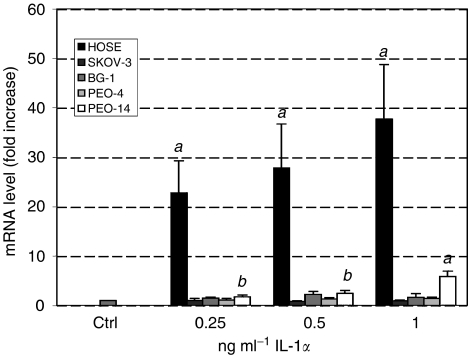
Real-time PCR of 11*β*HSD-1 in HOSE, SKOV-3, BG-1, PEO-4 and PEO-14 cells treated with increasing concentrations of IL-1*α*. Level of mRNA was standardised to 18S using an internal control and normalised to untreated cells. Data (*n*=6) are presented as mean±s.e.m. Letters denote significance above control: *a*: *P*<0.05 and *b*: *P*<0.01.

Treatment of HOSE cells with IL-1*α* dramatically enhanced the level of 11*β*HSD-1 mRNA up to 28-fold (*P*<0.01) in a dose-dependant fashion (Figure 3). In contrast, with the exception of PEO-14 cells, IL-1*α* did not alter the level of 11*β*HSD-1 mRNA in the other cell lines tested. For PEO-14 cells, a maximal three-fold induction of 11*β*HSD-1 mRNA was observed in response to 1 ng ml^−1^ IL-1*α* (*P*<0.01).

### Effect of IL-1*α* on 11*β*HSD-2 mRNA in cancer cell lines and HOSE cells

**Figure 4:**
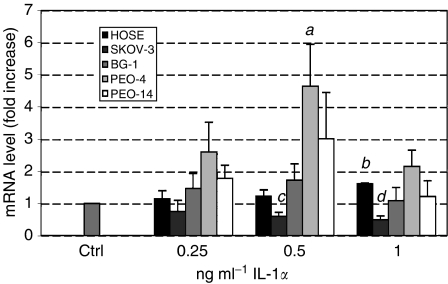
Real-time PCR of 11*β*HSD-2 in HOSE, SKOV-3, BG-1, PEO-4 and PEO-14 cells treated with increasing concentrations of IL-1*α*. Level of mRNA was standardised to 18S using an internal control and normalised to untreated cells. Data (*n*=6) are presented as mean±s.e.m. Letters denote significance above control: *a*: *P*<0.05 and *b*: *P*<0.001, and below control: *c*: *P*<0.05 and *d*: *P*<0.01.

The level of 11*β*HSD-2 mRNA in HOSE cells was unaffected by the presence of IL-1*α* except for marginal stimulation in response to 1 ng ml^−1^ IL-1*α* (1.6-fold; *P*<0.001), see Figure 4. The greatest stimulation of 11*β*HSD-2 mRNA level was observed in PEO-4 cells treated with 0.5 ng ml^−1^ IL-1*α* (4.6-fold; *P*<0.05). For BG-1 and PEO-14 cells, 11*β*HSD-2 mRNA level was similarly observed to increase in response to IL-1*α*; however, this was not significant. In contrast, addition of IL-1*α* to SKOV-3 cells induced a significant decrease of 11*β*HSD-2 mRNA level (two-fold; *P*<0.01).

### Effect of IL-1*α* on COX-2 mRNA in cancer cell lines and HOSE cells

**Figure 5:**
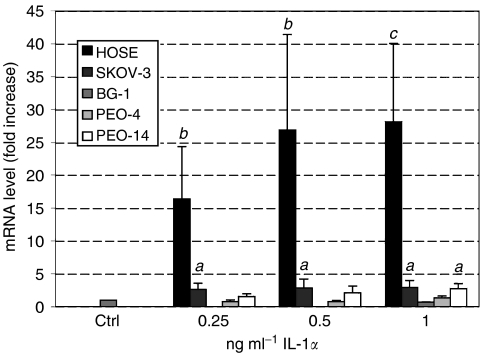
Real-time PCR of COX-2 in HOSE, SKOV-3, BG-1, PEO-4 and PEO-14 cells treated with increasing concentrations of IL-1*α*. Level of mRNA was standardised to 18S using an internal control and normalised to untreated cells. Data (*n*=6) are presented as mean±s.e.m. Letters denote significance above control: *a*: *P*<0.05, *b*: *P*<0.01 and *c*: *P*<0.001.

Treatment of HOSE cells with IL-1*α* dramatically increased the level of COX-2 mRNA in a dose-dependant fashion exhibiting maximal stimulation with 1 ng ml^−1^ IL-1*α* (28-fold; *P*<0.001), see Figure 5. In contrast, the level of COX-2 mRNA in the cell lines was largely unaffected; only a mild stimulation was observed in response to 1 ng ml^−1^ IL-1*α* in either SKOV-3 or PEO-14 cells (2.8-fold, *P*<0.05).

## DISCUSSION

This study reveals unique immuno-endocrine signatures for four ovarian adenocarcinoma cell lines (SKOV-3, BG-1, PEO-4 and PEO-14) that clearly distinguish them from the nontransformed HOSE cell type from which most ovarian cancers are presumed to derive. Whereas primary HOSE cell cultures show highly IL-1*α* responsive COX-2 and 11*β*HSD-1 gene expression, neither gene in the cancer cell lines responded markedly to IL-1*α*. In contrast, whereas HOSE cells exhibited a low level of 11*β*HSD-2 mRNA that was mildly responsive to IL-1*α* (1.6-fold, *P*<0.001), all cell lines exhibited a higher basal level (*P*<0.01) of 11*β*HSD-2 mRNA that was in some cases further stimulated in PEO-4 cells (five-fold; *P*<0.05) or suppressed in SKOV-3 cells (two-fold; *P*<0.01), in response to IL-1*α*. Increased expression of COX-2 is a hallmark of the inflammatory response. The stimulation of COX-2 in HOSE cells (28-fold, *P*<0.001) and relative lack of stimulation in SKOV-3, BG-1 and PEO-4 cancer cell lines, in response to IL-1*α*, therefore, suggests that these cancer cell lines have lost the ability to induce a normal inflammatory response. It remains to be determined if these findings translate to primary ovarian cancer cells; however, we consider the data presented here to justify such an analysis if the ethical and logistical constraints in establishing suitable primary cell cultures from sufficient patients can be overcome.

Based on the divergent effects of 11*β*HSD-1 and -2 on cell proliferation observed *in vitro*, Rabbitt *et al* (2003) have previously suggested that the ability of 11*β*HSD-1 to generate cortisol might act as an autocrine antiproliferative, prodifferentiation stimulus in normal adult tissues. In contrast, the cortisol-inactivating properties of 11*β*HSD-2 might lead to pro-proliferative effects, particularly in tumours. Our finding that normal OSE cells, unlike cell lines derived from ovarian tumours, abundantly express 11*β*HSD-1 mRNA but are essentially devoid of 11*β*HSD-2 mRNA, supports the concept that the pattern of 11*β*HSD isoform expression is a defining feature of neoplastic cellular transformation that could have particular relevance to the ovary.

Based on the level of IL-1R mRNA, the differential effects observed in response to IL-1*α* between cell lines and HOSE cells may simply result from differential IL-1R expression. Although IL-1R mRNA is more abundant in HOSE cells and PEO-14 cells, IL-1R mRNA is nevertheless detectable in the other cell lines tested (Figure 2). The observation that the level of 11*β*HSD-2 mRNA is more greatly increased in response to IL-1*α* in cancer cell lines relative to HOSE cells (Figure 4) may suggest that expression of IL-1R is not limiting the effects of IL-1*α* on gene expression in the cancer cell lines. In the case of SKOV-3, IL-1R signalling was previously shown to be functional by demonstrating activation of NF-kappa in response to IL-1*β* (Bonizzi *et al*, 1996).

IL-1R signalling is initiated by receptor-associated proteins that include TRAFs (TNF receptor-associated factors; Inoue *et al*, 2000), TIRs (Toll IL-1 receptor proteins; McGettrick and O'Neill, 2004) and IRAKs (IL-1 receptor-associated kinases; Janssens and Beyaert, 2003). IL-1 also activates MAP kinase pathways: p38 (Guan *et al*, 1997), ERK 1/2 ((Laporte *et al*, 1999) and JNK (Guan *et al*, 1998) and subsequently activates transcription factors such as NF-k*β* (Roshak *et al*, 1996). In addition, modulation of IL-1R signalling is altered by other signalling proteins such as Smads, SOCSs (suppressor of cytokine signalling; Yoshimura *et al*, 2003) and STATs (Morton *et al*, 1999). In one study of ovarian carcinoma, SOCS 1 and 2 genes were demonstrated to be silenced due to hypermethylation (Sutherland *et al*, 2004) and a number of studies have identified increased levels of STAT proteins (particularly STAT 3) in ovarian cancers (Huang *et al*, 2000; Chen *et al*, 2004; Meinhold-Heerlein *et al*, 2005; Silver *et al*, 2004). We are currently focusing our attention on IL-1R signalling in order to identify any differences between primary HOSE cells and ovarian cancer cells.

Although there are clearly differences between all cell lines and HOSE cells, the PEO-14 cell line appears most closely to resemble HOSE cells. Relative to SKOV-3, BG-1 and PEO-4 cells, PEO-14 and HOSE cells express higher levels of 11*β*HSD-1, COX-2 and IL-1R mRNA and lower levels of 11*β*HSD-2 mRNA. Moreover, whereas 11*β*HSD-2 mRNA is unaltered in response to IL-1*α*, the level of 11*β*HSD-1 and COX-2 mRNA is stimulated in response to IL-1*α* in both HOSE and PEO-14 cells. The observation that the PEO-14 cell line most closely resembles HOSE cells is consistent with the origin of PEO-14 cells from a well-differentiated serous adenocarcinoma; SKOV-3, BG-1 and PEO-4 cells originate from poorly differentiated serous adenocarcinoma. Since the cell lines used in this study are derived from different ovarian cancers at different stages of differentiation, it is possible that the heterogeneity observed is reflective of these differences. Indeed, the responses observed may provide a novel means of defining such differences at a functional level.

Whereas 11*β*HSD-1 predominantly catalyses 11-oxoreduction of cortisone to cortisol, 11*β*HSD-2 converts cortisol to cortisone. The pattern of 11*β*HSD type 1 and 2 expression described in HOSE cells is therefore consistent with an increased conversion of cortisone to cortisol upon exposure to IL-1*α*, previously identified in HOSE cells (Yong *et al*, 2002). Thus, upregulation of 11*β*HSD-1 without a measurable change in 11*β*HSD-2 would be predicted to result in increased conversion of systemically derived cortisone to anti-inflammatory cortisol in the OSE. This is proposed as a compensatory anti-inflammatory mechanism that accompanies the inflammatory response (e.g. COX-2 expression) to cytokines shown by the OSE during LH-induced ovulation *in vivo*.

Receptors for glucocorticoids are present in tumour cells of almost 90% of ovarian cancers, and these hormones inhibit ovarian cancer cell growth (Karlan *et al*, 1994). It is therefore of interest that three epithelial cancer cell lines (SKOV-3, BG-1 and PEO-4) exhibit a low basal level of 11*β*HSD-1 that is unresponsive to inflammatory stimulation, yet have gained a basal level of 11*β*HSD-2 mRNA that, in the case of the PEO-4 cell line, is cytokine responsive. For HOSE cells, addition of cortisol augments IL-1*α* stimulation of 11*β*HSD-1, but decreases IL-1*α* induced COX-2 expression (Rae *et al*, 2004). The increased level of cortisol, in response to IL-1*α* induced 11*β*HSD-1, is thereby envisaged to function in an anti-inflammatory manner. The impact of glucocorticoids on IL-1*α* responsiveness in ovarian cell lines remains to be assessed; however, it is interesting to note that unlike HOSE and other cell lines, the BG-1 cell line does not appear to express GR*α*.

The potential pathophysiological relevance of these findings relates to the likelihood that serial inflammatory injury associated with ovulation predisposes the OSE to neoplastic transformation. Factors related to inflammation of the OSE have been associated with increased risk of ovarian cancer (Ness and Cottreau, 1999; Ness *et al*, 2000) and exposure to anti-inflammatory agents has been shown to inhibit tumour invasion and protease production by ovarian carcinoma cells (Akhmedkhanov *et al*, 2001). It is therefore of obvious interest that all cell lines studied here appear to have largely lost their capacity to mount an inflammatory response to IL-1*α* in terms of increased 11*β*HSD-1 expression and hence increased formation of anti-inflammatory, proapoptotic glucocorticoids. It remains to be determined whether this loss of response to proinflammatory cytokines is a feature of the primary tumours from which ovarian cancer cell lines are derived and whether it might be causal or consequential to disease progress.
